# GhMCS1, the Cotton Orthologue of Human GRIM-19, Is a Subunit of Mitochondrial Complex I and Associated with Cotton Fibre Growth

**DOI:** 10.1371/journal.pone.0162928

**Published:** 2016-09-15

**Authors:** Chun-Juan Dong, Ai-Min Wu, Shao-Jun Du, Kai Tang, Yun Wang, Jin-Yuan Liu

**Affiliations:** Laboratory of Plant Molecular Biology, Center for Plant Biology, School of Life Sciences, Tsinghua University, Beijing, China; USDA-ARS Southern Regional Research Center, UNITED STATES

## Abstract

GRIM-19 (Gene associated with Retinoid-Interferon-induced Mortality 19) is a subunit of mitochondrial respiratory complex I in mammalian systems, and it has been demonstrated to be a multifunctional protein involved in the cell cycle, cell motility and innate immunity. However, little is known about the molecular functions of its homologues in plants. Here, we characterised GhMCS1, an orthologue of human GRIM-19 from cotton (*Gossypium hirsutum* L.), and found that it was essential for maintaining complex integrity and mitochondrial function in cotton. *GhMCS1* was detected in various cotton tissues, with high levels expressed in developing fibres and flowers and lower levels in leaves, roots and ovules. In fibres at different developmental stages, *GhMCS1* expression peaked at 5–15 days post anthesis (dpa) and then decreased at 20 dpa and diminished at 25 dpa. By Western blot analysis, GhMCS1 was observed to be localised to the mitochondria of cotton leaves and to colocalise with complex I. In Arabidopsis, *GhMCS1* overexpression enhanced the assembly of complex I and thus respiratory activity, whereas the *GhMCS1* homologue (*At1g04630*) knockdown mutants showed significantly decreased respiratory activities. Furthermore, the mutants presented with some phenotypic changes, such as smaller whole-plant architecture, poorly developed seeds and fewer trichomes. More importantly, in the cotton fibres, both the *GhMCS1* transcript and protein levels were correlated with respiratory activity and fibre developmental phase. Our results suggest that GhMCS1, a functional ortholog of the human GRIM-19, is an essential subunit of mitochondrial complex I and is involved in cotton fibre development. The present data may deepen our knowledge on the potential roles of mitochondria in fibre morphogenesis.

## Introduction

Mitochondria in plants, similar to other eukaryotes, are highly specialised and dynamic double membranous organelles that play a pivotal role in energy production through oxidative phosphorylation (OXPHOS) [1]. The OXPHOS system is localised to the mitochondrial inner membrane, and it is composed of five complexes (I to V) that work in concert to drive the aerobic synthesis of ATP [2]. Of these complexes, complex I or NADH:ubiquinone oxidoreductase (E.C. 1.6.5.3) is the first and largest enzyme of the OXPHOS system that contains over 40 different subunits in eukaryotes, including mammals and plants [3–5]. It catalyses the transfer of electrons from NADH to ubiquinone in a process coupled to proton transport across the mitochondrial inner membrane. Obviously, the maintenance of its integrity and function depends on the contribution of all of the proteins that are encoded by both the nuclear and mitochondrial genomes. It has been reported that its defect in complex I is the most common cause of mitochondrial disorders, leading to disruption of cell function and subsequent disease or even death [4, 6]. Mutations have been found in both mitochondrial and nuclear genes, such as *GRIM-19* (gene associated with retinoid-interferon-induced mortality-19), which leads to the complete abolishment of complex I assembly when deleted; additionally, its knockout causes early embryonic lethality in mice [4, 7].

Strikingly, although *GRIM-19* was initially identified as an interferon-β and retinoic acid-inducible gene with apoptotic effects in human cancer cell lines [7], recent studies have demonstrated that GRIM-19 is a nuclear-encoded subunit of complex I that is critical for assembly and enzymatic activity of mitochondrial complex I in mice [8]. Further, it has been shown that GRIM-19 is involved in the cell cycle, cell motility and innate immunity in mammalian cells [9, 10]. Therefore, it appears to be a multifunctional protein involved in cell death, maintenance of mitochondrial metabolism, growth regulation and innate immunity. Although this protein has been studied in humans and other mammals, as well as in some insects and fish [11, 12], it has not been described in plants to date.

The cotton (*Gossypium* spp.) fibre consists of a single epidermal cell derived from the ovule, and it plays a significant role in the global textile industry [13–15]. After differentiation (prior to the day of anthesis), the emerged fibre cells on the surfaces of cotton ovules undergo rapid elongation [5–20 days post anthesis (dpa)], secondary cell wall synthesis (15–45 dpa) and dehydration (40–60 dpa), finally forming mature long fibres that are harvested from seeds and used in textile products [13, 16, 17]. These economically important single cells dramatically elongate after anthesis, typically reaching up to 30 mm in length in most commercially important species. Therefore, the fibre cells can serve as the excellent single-celled model system for studying cell differentiation and growth [13, 18].

It has been well-established that various fibre-specific and fibre-enriched genes are involved in the different stages of cotton fibre development, such as cellulose synthase [19], endo-1,4-β-glucanase [20], sucrose synthase [21], α- and β-tubulin [22, 23], reversibly glycosylated polypeptide [24], β-galactosidase [25], glucuronosyltransferase [26], actin [27], the MYB transcription factors [28, 29], phosphatise and annexin [30, 31]. Over the past decade, genome-wide transcriptomic and proteomic studies have increased the understanding of the molecular basis of fibre development [13, 14, 18, 32–34]. These studies have revealed that many important molecular processes, such as glycolysis, the tricarboxylic acid (TCA) cycle, redox homeostasis, cytoskeleton dynamics and anthocyanidin metabolism, are differentially regulated during fibre initiation and elongation. Among these processes, the TCA cycle, which is one of the three main stages of cellular respiration, occurs in the matrix of the mitochondrion, while OXPHOS is the final step of cellular respiration that results in the production of ATP molecules [4]. Thus, it is well known that OXPHOS is critical to the integrity of fibre mitochondrial function and even to the development and growth of cotton fibre cells. However, there are few reports of the potential influences of some components of the cotton fibre mitochondrial OXPHOS system on the integration of major fibre metabolism as well as fibre cell growth.

To investigate the function of the cotton orthologue of human GRIM-19, the full-length cDNA sequence of *GhMCS1* was cloned from cotton fibre cells and characterised. *GhMCS1* is highly expressed in developing fibres and flowers and exhibits a specifically temporal expression pattern correlating with fibre growth. Biochemical detection of GhMCS1 showed its mitochondrial localisation and key role in complex I assembly in cotton mitochondria. *GhMCS1* expression was closely correlated with fibre mitochondrial activity and development, which indicates that GhMCS1, as a subunit of mitochondrial complex I, is essential for mitochondrial function and cotton fibre morphogenesis.

## Materials and Methods

### Plant materials

Upland cotton (*Gossypium hirsutum* L. cv. CRI35) was grown in the field under standard conditions at the Tsinghua University in China as described previously [35]. The seeds were kindly provided by the Cotton Research Institute, Chinese Academy of Agricultural Science. When cotton plants were in full bloom (approximately 90 days after planting), we collected different cotton tissues, including roots, stems, leaves, flowers, and intact ovules. Cotton fibers were harvested at 5, 10, 15, 20, 25, 30, 35, and 40 days post anthesis (dpa). All of these samples were immediately frozen in liquid nitrogen and then stored at -80°C until extraction.

All Arabidopsis plants, including two mutant lines of the Col genetic background, *At1g04630*.*1* (SALK_020599C) and *At1g04630*.*2* (CS26138), were grown in fully automated growth chambers as described previously [36].

### Cloning of *GhMCS1* gene

Genomic DNA was extracted from young cotton leaves with a DNeasy Plant Kit (Qiagen, Valencia, CA, USA). Total RNA was isolated from cotton seedlings and various tissues using the TRIzol Reagent (Invitrogen, Carlsbad, CA, USA) following purification using an RNeasy Plant Mini Kit (Qiagen, Valencia, CA, USA). For the samples of 30~40-dpa fibres, before purification with the kit, the crude RNA was precipitated in 1/3 volume 8M LiCl at 4°C overnight to remove the contaminants (eg. sugars, phenols, etc.) [37]. First-strand cDNA synthesis was performed with Superscript III reverse transcriptase (Invitrogen, Carlsbad, CA, USA).

A rapid amplification of cDNA ends (RACE) system (SMART^TM^ RACE cDNA Amplification Kit, Clontech, Palo Alto, USA) was used to isolate the *GhMCS1* cDNA. The gene-specific primers P1 (5′-CTTCACACTCGCCATTCCT-3′) and P2 (5′-GTCACTGTTGAGCTGTTTTCAC-3′) were designed to target the known *GhMCS1* sequence fragment and were used for the 5′-RACE and 3′-RACE assays, respectively. The PCR-amplified products were cloned into a pMD18-T vector (TakaRa, Tokyo, Japan) and sequenced. According to the RACE results, the primers FP1 (5′-ATGACTGAGGCAGTGATAAG-3′) and FP2 (5′-TCACCAAACTTCAGGCG-3′) were designed and used to amplify the full-length cDNA with a cotton cDNA library as the template.

The full-length genomic *GhMCS1* sequence was amplified from genomic DNA with TaKaRa LA Taq DNA polymerase and the FP1 and FP2 primers. Then, the PCR products were cloned into a pMD18-T vector (TakaRa, Tokyo, Japan) and sequenced. The promoter region of *GhMCS1* was cloned with an improved PCR-based genomic walking method described previously [26]. The PCR was performed with the following primers: an outer adaptor primer (AP1) (5′-GTAATACGACTCACTATAGGGC-3′) and an outer gene-specific primer (GSP1) (5′-CAAGAATCAGCAGAATCCTCCAC-3′). An inner adaptor primer (AP2) (5′-ACTATAGGGCACGCGTGGTC-3′) and an outer gene-specific primer (GSP2) (5′-GGCAAGGACCTGCGGATC-3′) were used for the second round of PCR. The major bands were isolated and sequenced. Finally, the 714-bp promoter region was amplified from the cotton genomic DNA using the primers pro-P1 (5′-AATACCGGGGATCCTCTAGA-3′) and pro-P2 (5′-CTGCCCTTGTTTTTCTGTATCG-3′).

### RNA analysis

For northern blot, 30 μg of total RNA were size fractionated on 1.2% agarose MOPS-formaldehyde gels and then transferred to Hybond-N^+^ nylon membrane (Amersham, Piscataway, NJ, USA) and hybridised with *GhMCS1* full-length cDNA labelled with [α-^32^P]dCTP as described previously [36].

Semi-quantitative RT-PCR was performed with specific primers using an RNA PCR kit (AMV, version 3.0) (TakaRa, Tokyo, Japan). PCR products were visualised with ethidium bromide staining. The primers used here were as follows: FP1 and FP2 for *GhMCS1*, At-P1 (5′-GATCGATTTGGGGATCAATCG-3′) and AtP2 (5′-CAATACATGAACTGTGTATGTATC-3′) for *At1g04630*, and At-P3 (5′-GGTCGAGAAGAAATTGAGCG-3′) and At-P4 (5′-CAAGAACATATCAATCCTCAGC-3′) for *At2g33220*.

### Generation of Arabidopsis transgenic plants

To produce Arabidopsis transgenic plants overexpressing *GhMCS1*, the *Xho* I-*Spe* I fragment of the *GhMCS1* cDNA was ligated into a plant binary vector, pTA7002, allowing for the DEX-inducible expression of *GhMCS1* [38]. The plants carrying the empty pTA7002 vector was used as a control. For the *PGhMCS1*::*GUS* construct, the 1,714-bp *Hin*d III-*Xba* I fragment of the *GhMCS1* promoter was ligated with the *GUS* reporter gene and then inserted into the corresponding sites of a pBE2113 vector to replace the CaMV35S promoter, producing a *pGhMCS1*::*GUS* plasmid. The recombinant plasmids were introduced into *Agrobacterium tumefaciens* GV3101 by the liquid nitrogen freeze-thaw method [39] and then transferred into Arabidopsis plants by the floral dip method as described previously [40, 41]. Homozygous plants were selected from the T_2_ progenies and confirmed in the T_3_ generations on the basis of hygromycin or kanamycin resistance. These homozygous plants were utilised in the described experiments.

### Subcellular localisation of GhMCS1 protein

Young leaves from 20-d-old cotton or Arabidopsis seedlings were used for isolation of subcellular fractions as described previously [42, 43]. Briefly, fresh leaves were washed in distilled water and gently homogenised with a mortar and pestle in extraction buffer (450 mM sucrose, 15 mM MOPS-KOH, pH 7.4, 3 mM EDTA, 0.2% BSA, 0.6% PVP-40, 10 mM DTT, and 0.2 mM PMSF). The homogenate was first filtered through 4 layers of pledget to remove large contaminants, small cell debris and nuclei by centrifugation at 100*×g* for 5 min. The supernatant was resuspended and served as the total protein.After recentrifugation at 3,500×*g* for 10 min to pellet crude chloroplasts, the supernatant was centrifuged at 17,000×*g* for 15 min to pellet mitochondria. The final supernatant was regarded as the cytosol fraction. This final pellet was resuspended with wash buffer (300 mM sucrose, 15 mM MOPS-KOH, pH 7.5, 3 mM EDTA, and 0.2 mM PMSF) and carefully pipetted onto the surface of a 40%/23%/18% Percoll step gradient. After centrifugation at 40,000*×g* for 45 min, the mitochondria appeared as a brown band at the surface of the 40%/23% Percoll layers, while the chloroplasts appeared as a bright yellow band at the 23%/18% Percoll interface. The subcellular fractions were carefully transferred to fresh tubes and washed with the wash buffer.

For immunolocalisation, the intact mitochondria, chloroplast and cytosol fractions were separated by SDS-PAGE and subjected to Western blot analysis. Western blot was performed as described previously [40], using anti-GhMCS1 serum as the probe for the GhMCS1 protein, while antibodies directed against the wheat subunit 9 of NADH dehydrogenase (NAD9) [42] and Arabidopsis photosystem II core protein CP43 [44] were used as controls for the mitochondrial and chloroplast fractions, respectively. To determine the submitochondrial localisation of GhMCS1, the mitochondrial samples were treated with proteinase K (100 ng/ml) for various durations in the absence or presence of 0.5% Triton X-100. Then, the mitochondrial proteins were subjected to SDS-PAGE and Western blot.

BN-PAGE was used for separation of different mitochondrial complexes on a 5 to 18% polyacrylamide gradient [45]. The respiration complexes were observed directly in the gel and were also subjected to Western blot analysis using antibodies against GhMCS1 or NAD9.

### Respiratory activity assay

Respiratory activity was determined as reported previously with some modifications [46]. Briefly, the fresh plant samples were incubated for 8 h in 5.0 ml of 2,3,4-triphenyl tetrazolium chloride (TTC, 0.1%) at 30°C in the dark. After incubation, the samples were washed with sterilized water for three times and then grinded. The formazan was extracted with 3.0 ml of acetone for 5 min. The optical density at 485 nm (*A*_485_) of the acetone solution was measured spectrophotometrically, the fresh weights (FW) of the samples were determined, and the *A*_485_ per gram FW was calculated as respiratory activity. The samples without TTC incubation were used as negative control.

### Statistical analyses

All data are expressed as the mean ± sd. Statistical analysis was performed with a two-tailed, unpaired, Student’s t-test. All differences were considered significant at *P*<0.05.

## Results

### Sequence characterization of *GhMCS1*

**Fig 1:**
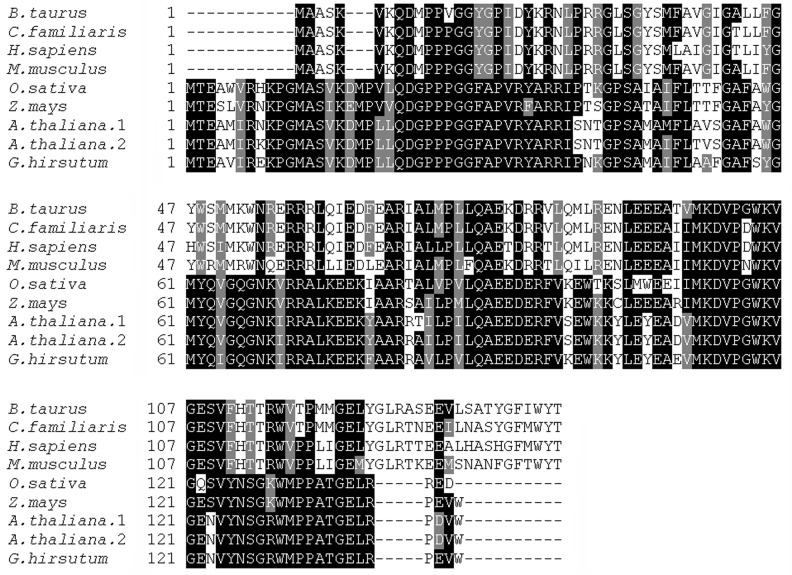
Alignments of the amino acid sequences of the GhMCS1 protein, the GRIM-19-like proteins from *Arabidopsis thaliana* (At1g04630 and At2g33220), rice (*Oryza sativa*, ABF94411), maize (*Zea mays*, NP_001148415), and the GRIM-19 proteins from bovine (*Bos taurus*, Q95KV7), dog (*Canis familiaris*, XP_533863), human (*Homo sapiens*, Q9P0J0), and mouse (*Mus musculus*, Q9ERS2). The sequences were aligned with Clustal W using the default parameters, and the box shading was created by BOXSHADE 3.21 (http://www.ch.embnet.org/software/BOX_form.html).

In our previous study, a novel cDNA has been detected to be preferentially expressed in developing fibre cells of upland cotton (GenBank Accession No. AJ311660) by fluorescence differential display PCR [24]. Partial amino acid sequence alignment has indicated that it shares a high level of sequence similarity with human GRIM-19. In the present study, we cloned its full-length cDNA sequence by the RACE-PCR and designated it as *GhMCS1* (*G**ossypium **h**irsutum* mitochondrial complex I subunit 1). By mapping to the NBI-Gh genome database (http://www.cottongen.org), the *GhMCS1* gene was matched to the Gh_A10G1065. As shown in S1 Fig, the open reading frame of *GhMCS1* contained 432 bp, encoding a protein of 143 amino acids with a predicted molecular mass of 16 kD and a theoretical p*I* of 9.83. Nuclear DNA sequence analysis illustrated that it was interrupted by two introns and that the first (4,844 bp) was much larger than the second (90 bp). The sequences surrounding the exon-intron junctions in the *GhMCS1* gene conformed to the “GT-AG” rule for donor/acceptor sites (S1 Fig) [47]. This gene structure was greatly similar to those of its homologues from other plant species, including Arabidopsis and rice. The long intron (4844 bp) indicated the possibility of alternative splicing. However, no alternative transcript was detected in the RT-PCR sequencing, suggesting that alternative splicing might not occur during *GhMCS1* transcription, or the expression of alternative transcripts was too low to be detected. BLAST analysis indicated that the deduced GhMCS1 protein shared at least 50% amino acid sequence homology with GRIM-19 homologues from animals and plants, including human, bovine, dog, mouse, maize, rice and Arabidopsis (Fig 1). In particular, GhMCS1 was highly similar to two GRIM-19-like protein homologues in Arabidopsis (At1g04630 and At2g33220) that shared 87.94% and 88.65% identities, respectively (Fig 1). Collectively, this sequence information suggests that this gene may encode a GRIM-19-like protein in cotton.

To test whether *GhMCS1* was present as multiple copies in tetraploid cotton, we searched the genome sequence of *h**irsutum* (AADD) using GhMCS1 as query [48]. Totally, we found two copies in A subgenome and D subgenome (S2 Fig). All of the four copies showed very high similarities with GhMCS1 in amino acid sequence and gene structure, although some SNP differences were detected (S2 Fig).

### Spatial and temporal expression patterns of *GhMCS1*

**Fig 2:**
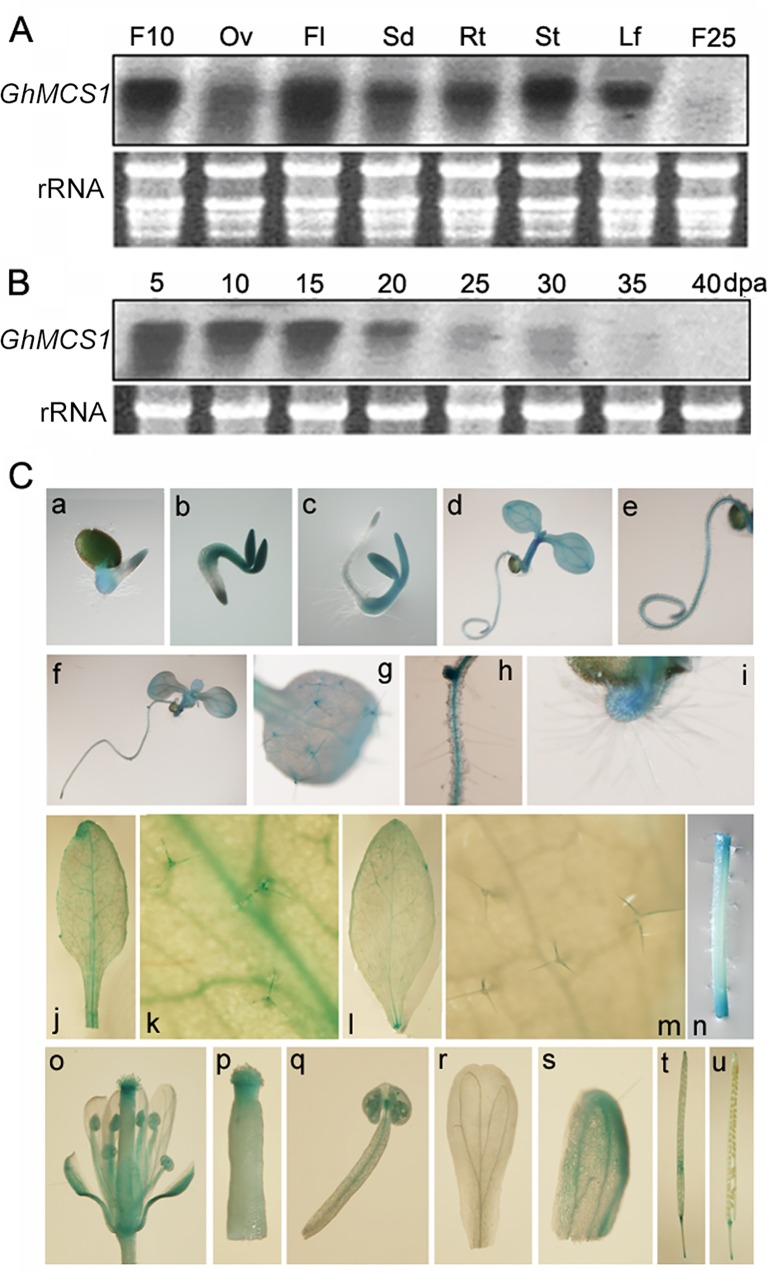
Spatial and temporal pattern of *GhMCS1* expression. (A) Northern blot analysis using RNA from seedlings (14 d old), flowers (0 dpa), developing ovules (10 dpa), and fibres. Total RNA was isolated from young true leaves (Lf), stems (St), roots (Rt), flowers (Fl), intact ovules (Ov), and fibre cells at 10 dpa (F10) and 25 dpa (F25). (B) Expression of *GhMCS1* in developing fibres at 5, 10, 15, 20, 25, 30, 35, 40 dpa, respectively. (C) Expression of the *pGhMCS1*::*GUS* reporter gene in transgenic Arabidopsis. (a-e) Seedlings grown on MS plates on day 1 (a), day 2 (b), day 3 (c) and day 5 (d), and magnification of root (e). (f-i) Seedlings on day 1 (f) and a magnified true leaf (g) and root (h-i). (j-m) Different leaf types of the transgenic plants. (j) A rosette leaf on day 20; (l) A cauline leaf on day 35; and (k, m) magnifications of j and l, respectively. (n) A stem. (o-u) Reproductive organs of the transgenic plants (o, mature flower; p, stigma; q, stamen; r, petal; s, sepal; t, young silique and u, old silique).

To examine the tissue-specific expression patterns of *GhMCS1*, Northern blot analysis was performed using a probe specific for the *GhMCS1* gene. As shown in Fig 2A, *GhMCS1* transcripts were detected at significantly high levels in the fibre cells compared with the naked ovules at 10 dpa. These results are consistent with our previous results [24]. In addition, *GhMCS1* transcripts were also observed in the leaves, stems and roots, with the highest levels occurring in the flowers at 0 dpa, suggesting that *GhMCS1* possessed constitutive expression patterns. During the fibre morphogenetic processes, *GhMCS1* transcript abundance was maintained at a high level in the rapidly elongating fibre cells (5–15 dpa), but it decreased significantly at 20 dpa (Fig 2B). *GhMCS1* mRNA was almost undetectable at 25 dpa and diminished at 35 dpa (Fig 2B), indicating that the *GhMCS1* gene was predominantly expressed during the fibre elongation stage but not during the subsequent secondary cell wall synthesis stage. It should be noted that because of the high sequence similarity and identical transcript sizes of the four *GhMCS1* copies, the spatial and temporal expression pattern is actually from all of four copies.

To gain a better understanding of the regulatory mechanism of the cotton *GhMCS1 g*ene, the 1,741-bp promoter sequence upstream of *GhMCS1* was cloned, and the promoter::GUS chimeric construct (*pGhMCS1*::*GUS*) was introduced into Arabidopsis. Histochemical GUS staining was performed in various organs throughout plant development. As shown in Fig 2C, GUS activity was detected in 1~3-d-old transgenic seedlings. GUS staining was strong in the cotyledons and hypocotyls but relatively weaker in the radicles; in which activity could be detected only at the root tips (Fig 2C and 2A–2C). In the 5- and 10-d-old seedlings, GUS expression was maintained in the cotyledons and hypocotyls (Fig 2C, 2D and 2F), but in the roots, only trace activity was present in the vascular tissues (Fig 2C, 2E, 2H and 2I). No activity was detected in the root hairs (Fig 2C, 2E, 2H and 2I). Interestingly, GUS expression was also observed in the new true leaves of 10-d-old seedlings, in which the trichomes could be stained (Fig 2C and 2G). Furthermore, in the rosette leaves of 20-d-old soil-grown seedlings, GUS activity could be detected only in the trichomes and veins (Fig 2C, 2J and 2K). In the cauline leaves of 35-d-old transgenic plants, a decreased number of trichomes was stained, and GUS activity was relatively weaker (Fig 2C, 2L and 2M). In the stems of soil-grown plants, no GUS activity could be detected except at the cutting sites (Fig 2C and 2N). The trichome and vascular tissues-preferential GUS activity further supported the notion that GhMCS1 was associated with the elongation of cotton fibres (Fig 2B).

To detect GUS expression in the reproductive organs of transgenic Arabidopsis plants, whole flowers and siliques were also stained (Fig 2C and 2O–2S), showing intense GUS activity in the stigmas (Fig 2C and 2P). In the stamens, staining could be observed in the anthers (Fig 2C and 2Q). GUS expression was also detected in the sepals (Fig 2C and 2S) but not in the petals (Fig 2C and 2R). In the young siliques, some weak GUS staining was observed (Fig 2C and 2T), which decreased in intensity as the siliques matured (Fig 2C and 2U). Considering the northern blot and histochemical GUS assay results, *GhMCS1* was highly expressed in the trichomes of the transgenic Arabidopsis plants and the elongating fibre cells, which are trichomes of cotton ovules, as well as in the flower tissues, and it is likely that it is precisely regulated during plant development.

### Mitochondrial localisation of GhMCS1 protein

**Fig 3:**
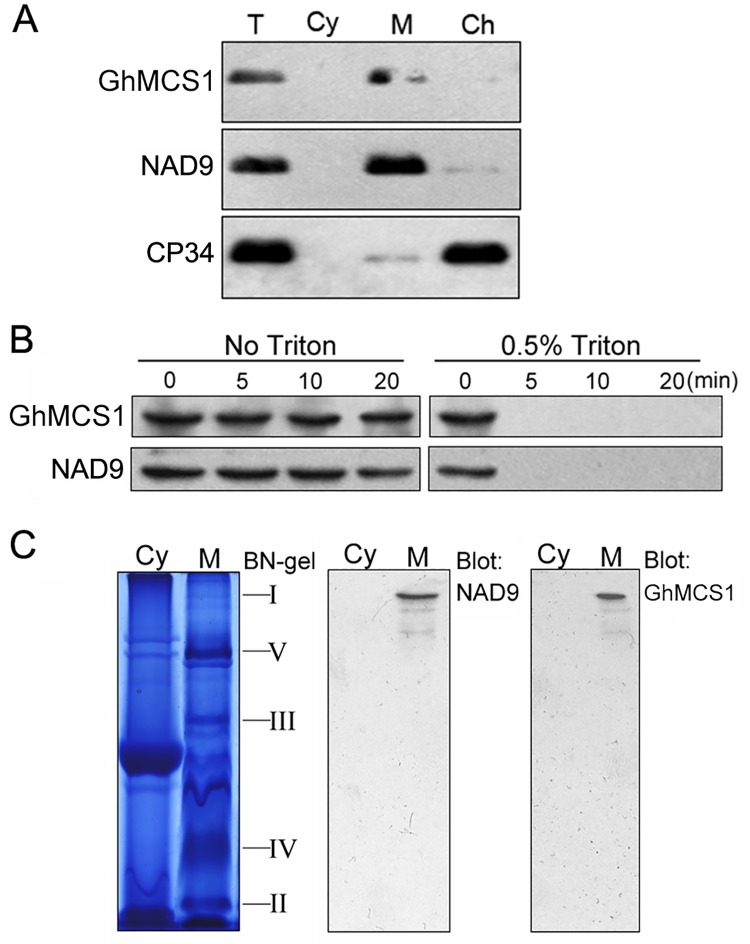
Subcellular localisation of GhMCS1. (A) Mitochondrial localisation of GhMCS1. Cotton leaves were lysed and divided into cytoplasmic (Cy), mitochondrial (M), and chloroplast (Ch) fractions. The expression of GhMCS1 in the total cell lysates (T, containing 50 μg of proteins) and different fractions (containing 25 μg of proteins in each) were examined by Western blot with antiserum against GhMCS1. The blots were also probed with anti-NAD9 as a mitochondrial marker and anti-CP34 as a chloroplast marker. (B) Submitochondrial localisation of GhMCS1. The isolated mitochondria were treated with 100 ng of proteinase K per ml for various durations in the absence or presence of 0.5% Triton X-100. Then, the proteins were separated by SDS-PAGE and subjected to Western blot analysis with antibodies against GhMCS1 and NAD9. (C) Detection of GhMCS1 in complex I by BN-PAGE. The isolated cytoplasm (Cy) and mitochondria (M) were separated by BN-PAGE and detected by Western blot. Complex I was detected using anti-NAD9. GhMCS1 was detected in complex I with an anti-GhMCS1 antibody. The portions of the complexes and GhMCS1 are indicated on the right.

It is well known that proteins must localise to their appropriate subcellular compartments to perform their desired functions. To examine the cellular localisation of GhMCS1, cotton leaves were lysed and divided into cytoplasmic, mitochondrial, and chloroplast fractions, and then GhMCS1 protein levels were detected by Western blotting with a rabbit polyclonal antibody against cotton GhMCS1. As shown in Fig 3A, GhMCS1 was detected in the mitochondrial but not in the chloroplast or soluble cytoplasmic fractions. Detection of the mitochondrial marker protein NAD9 in the mitochondrial fraction and the chloroplast marker protein CP34 in the chloroplast fraction indicated ideal fraction separation. The results presented here support the notion that GhMCS1 indeed localises to cotton mitochondria, similar to the GRIM-19 protein in animals [8].

As reported previously, mitochondrial outer membrane proteins, such as BCL2, maybe digested by proteinase K in the absence of Triton X-100, whereas COX IV localised to the inner membrane or Hsp60 localised to the mitochondrial matrix cannot be digested under the same conditions [8]. However, all three proteins are digested by proteinase K in the presence of Triton X-100 [8]. To investigate sub-mitochondrial localisation of GhMCS1 protein, the mitochondrial protein fraction was treated with proteinase K in the presence or absence of 0.5% Triton X-100. As shown in Fig 3B, the GhMCS1 protein and the NAD9 marker could not be digested by proteinase K within the various treatment durations, even after 20 min of treatment, in the absence of Triton X-100. In contrast, both proteins were digested after a 5 min treatment when mitochondria were disrupted in the presence of Triton-X 100. These results suggest that GhMCS1 is localised either to the inner membrane or matrix but not to the outer membrane of mitochondria.

To obtain direct evidence that GhMCS1 is a component of mitochondrial complex I in cotton, a Blue native (BN)-PAGE assay was performed together with Western blot analysis. Mitochondria were isolated from cotton leaves, and OXPHOS complexes were resolved with BN-PAGE. Five major bands representing the enzyme complexes I to V were observed in the gel (Fig 3C). The identity of the target complex was verified by Western blot analysis with corresponding antibodies. As shown in Fig 3C, complex I, which was the largest complex, was detected with the antibody against the mitochondrial marker NAD9 protein. More importantly, the same complex was also recognised by the anti-GhMCS1 antiserum, whereas the other complexes were not detected (Fig 3C), indicating that GhMCS1 is physically present in cotton mitochondrial complex I in its native form.

### Changes in mitochondrial activity in *GhMCS1*-expressing transgenic Arabidopsis

To further investigate its potential biological roles, a plasmid harbouring *GhMCS1* cDNA and a dexamethasone (DEX)-inducible promoter were introduced into Arabidopsis (S3 Fig) [37]. Assayed by semi-quantitative RT-PCR, six independent homozygous lines with high levels of transgene expression were obtained (S3 Fig). Three of these transgenic lines (lines 2, 11 and 13) were randomly selected for subsequent biochemical assays. For each transgenic line, the results of the Western blot showed that the GhMCS1 protein could be induced by DEX, and its levels increased as DEX concentrations increased from 0 to 10 μM (S3 Fig). All of the accumulated GhMCS1 was localised to the mitochondria, even in the presence of DEX concentrations as high as 10 μM (S3 Fig)

**Fig 4:**
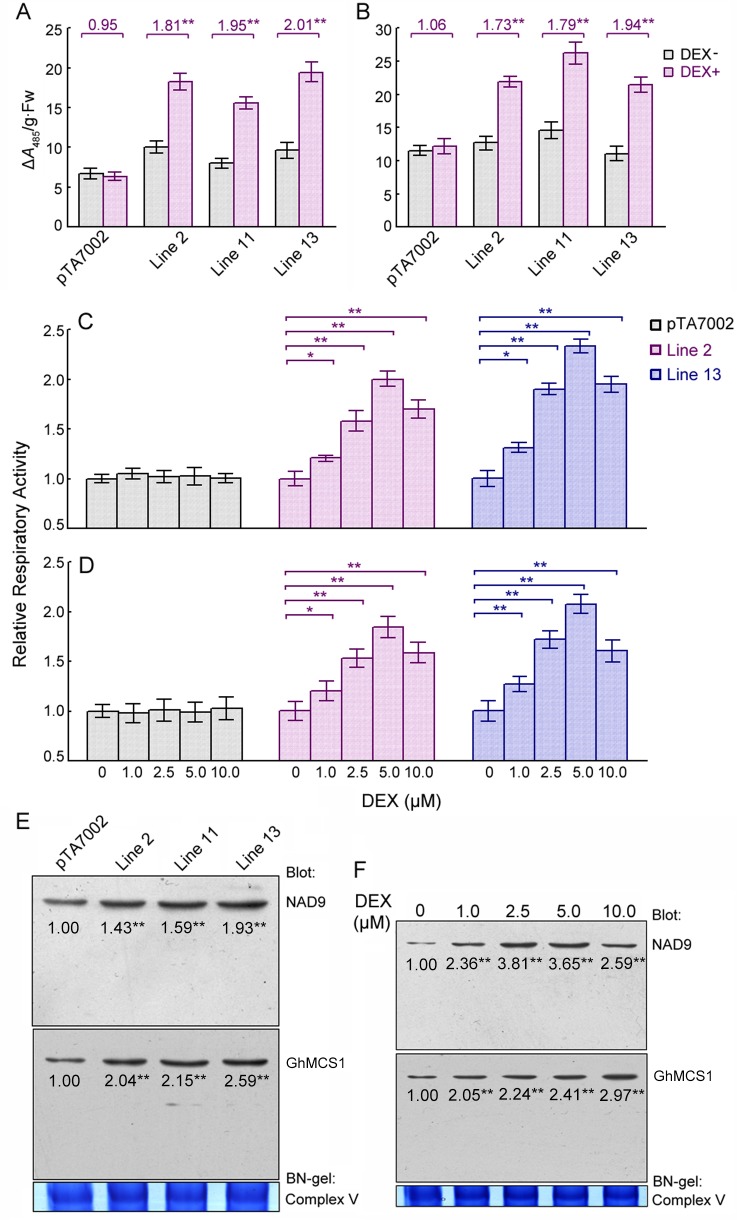
Respiratory activity and mitochondrial complex I assembly associated with *GhMCS1* expression in transgenic Arabidopsis plants. (A, B) Effects of GhMCS1 expression on respiratory activity in the shoots (A) or roots (B) of different transgenic lines. 10-d-old seedlings germinated and grown in the absence of DEX were transferred to inductive medium containing 5 μM DEX and cultured at 23°C for 3 d. The seedlings that had not been induced by DEX were used as controls. pTA7002 represents the Arabidopsis seedlings carrying the empty pTA7002 as negative controls. The numbers over the bars indicate the ratios of respiratory activity of the plants with and without DEX induction. (C, D) Dose dependencies of respiratory activity in the shoots (C) and roots (D) in transgenic lines 2 and 13. 10-day-old seedlings of different lines grown on non-inductive medium were transferred to medium containing each concentration of DEX and cultured at 23°C for 3 days. (E) Effects of GhMCS1 on the assembly ability of mitochondrial complex I. 10-day-old seedlings were lysed following 3 d of exposure to 5 μM of DEX, and the isolated mitochondria were subjected to BN-PAGE and assessed immunologically with anti-GhMCS1 and anti-NAD9. (F) Dose dependency of mitochondrial complex I assembly. Seedlings of the transgenic line 2 were used for mitochondrial isolation and Western blot analysis. Complex V was used as a loading control for the BN gel. The numbers under the bands indicate the relative abundance of NAD9 and GhMCS1 proteins. The relative abundance was statistically analyzed by BandScan 5.0. Error bars indicate the sd (n≥15) from three independent experiments. *, *P* < 0.05 and **, *P* < 0.01; both compared with the corresponding samples without DEX induction.

To examine the effects of *GhMCS1* expression on mitochondrial activity, respiratory activity was assayed in transgenic Arabidopsis seedlings without or with DEX induction. As shown in Fig 4, an approximate two-fold increase in respiratory activity was detected in both the shoots (Fig 4A) and roots (Fig 4B) of the transgenic plants compared to those of the control plants or non-DEX-induced seedlings. Further analysis revealed that respiratory activities increased with increasing DEX concentrations; however, the activities in both the shoots and roots declined when the DEX level reached 10 μM (Fig 4C and 4D). These decreases were presumably due to deficits in other mitochondrial components. The data presented here indicate that respiratory activity in plants is closely associated with GhMCS1 protein concentration within a certain range.

As described above, the GhMCS1 protein was identified to be an important component of cotton mitochondrial complex I (Fig 3). To further understand the effect of *GhMCS1* levels on mitochondrial activity, BN-PAGE was used together with Western blotting to analyse the assembly of intact multi-subunit complexes of the mitochondrial respiratory chain. As expected, a distinct increase in GhMCS1 protein abundance in mitochondrial complex I was detected in the *GhMCS1*-expressing transgenic Arabidopsis compared to the control plants harbouring empty vectors (Fig 4E). Strikingly, increased numbers of complex I could be detected by the anti-NAD9 antibody in the three transgenic lines (Fig 4E). Interestingly, a gradually increasing band representing complex I was observed along with increased levels of GhMCS1 (Fig 4F), indicating the efficient assembly of intact multi-subunit complexes of the mitochondrial respiratory chain in the transgenic Arabidopsis plants. Taken together, our data suggest that GhMCS1 physically resides in complex I and plays an essential role in the assembly of this complex and mitochondrial respiration. Nevertheless, no significant phenotypic differences were observed between the transgenic over-expressing plants with an approximate two-fold increase in respiratory activity and the control plants, neither during the early stage of vegetative growth nor during the reproductive stage (data not shown).

### Phenotypic changes and mitochondrial activity in Arabidopsis with *At1g04630* mutation

As indicated above, no distinct phenotypic changes were observed when GhMCS1 was overexpressed in Arabidopsis. One of the most direct approaches for assessing the function of a gene is to observe the effects on an organism that occur when that gene is missing (loss-of-function). To circumvent difficulties in regeneration of cotton plants, many fibre-specific or preferentially expressed genes or promoters have generally been analysed in model plants, such as Arabidopsis [28, 49] or tobacco [26]. As shown in Fig 1, GhMCS1 has two highly similar Arabidopsis homologues, At1g04630 and At2g33220, which shared 87.94% and 88.65% amino acid sequence identities with GhMCS1, respectively. As an initial step towards investigating biological function, their expression levels were examined. RT-PCR showed that the transcript levels of *At1g04630* and/or *At2g33220* in the leaves and stems were much higher than those in the roots (S4 Fig). In the reproductive tissues, the transcript levels were somewhat lower, and the levels in the flowers were higher than those in the siliques and seeds (S4 Fig). This expression pattern was very similar with that of *GhMCS1* in cotton (Fig 2A), especially the GUS distribution pattern in the transgenic Arabidopsis plants harbouring *pGhMCS1*::*GUS* (Fig 2C), suggesting that *At1g04630* (and/or *At2g33220*) presents with similar tissue distributions as GhMCS1 in cotton.

**Fig 5:**
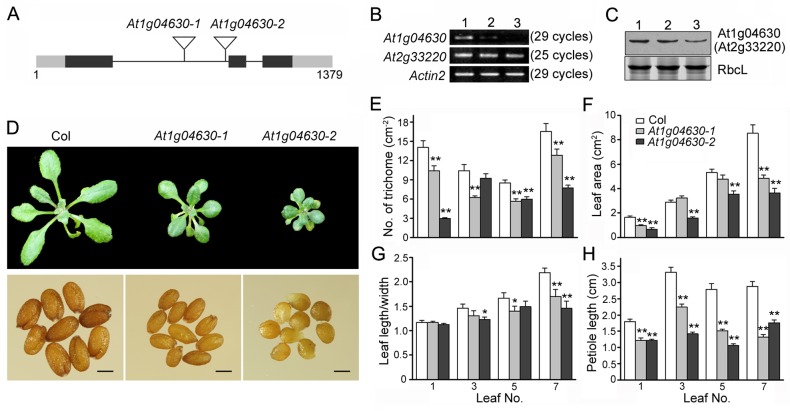
Loss-of-function mutants of *At1g04630* gene. (A) Schematic structures of the T-DNA insertion alleles, *At1g04630-1* and *At1g04630-2*. The black rectangles represent the exons, and the lines depict the introns. The grey rectangles represent the 5’- and 3’-UTRs. The triangles represent the T-DNA or transposon insertions. (B) Confirmation of the down-regulation of the *At1g04630* transcripts by RT-PCR. PCR was performed using primers targeting the 5’- and 3’-UTRs of *At1g04630* and *At2g33220* with 25 or 29 amplification cycles as indicated. Lane 1, wild type (Col); lane 2, *At1g04630-1*; lane 3, *At1g04630-2*. Arabidopsis *actin2* was amplified as a control. (C) Immunoblot analysis using an antibody against cotton GhMCS1. 10-d-old seedlings from each line were used for protein extraction. 15 mg of total protein were separated by SDS-PAGE and subjected to Western blot analysis. The large subunit of RuBisco (RbcL) was used as the loading control. (D) The differences in whole-plant phenotypes (25-d-old) and seed sizes between the wild type (Col) and mutant seedlings. The scale bar indicates 0.5 mm. (E) Quantification of trichomes in the rosette leaves of the wild types (Col) and *At1g04630* mutants. (F) The leaves possessed smaller areas in the mutants compared with the wild types. (G) The leaves were more rotund (smaller length:width ratio) in the mutants compared with the wild types. (H) Petiole lengths were shorter in the mutants than in the wild types. The different leaf phenotypes are shown in S4 Fig. Error bars indicate the sd (n = 15) from two independent experiments. *, *P*<0.05 and **, *P*<0.01; both compared with the wild type plants.

For loss-of-function analysis, Arabidopsis mutants with T-DNAs or transposons inserted into *At1g04630* and *At2g33220* were searched for in the ABRC stocks (Ohio State University, Columbus, OH). Fortunately, two homozygous mutant alleles, *At1g04630-1* (SALK_020599C) and *At1g04630-2* (CS26138) were obtained. For the *At2g33220* gene, a T-DNA-inserted mutant, SALK_054968, was found. However, no homozygous line was separated. We ultimately chose *At1g04630-1* and *At1g04630-2* for subsequent assays. As shown in Fig 5A, both mutants carried a T-DNA or transposon insertion in the first intron of *At104630* that separated the transcriptional from the translational start sites. RT-PCR analysis showed very low *At1g04630* transcript levels (Fig 5B), but no change in expression was found for the *At2g33220* mRNA (Fig 5B). Western blot analysis also showed that At1g04630 protein levels decreased significantly compared to those in the wild type plants, and in *At1g04630-2* mutant, the protein was expressed at the lowest levels (Fig 5C). In terms of phenotypes, both the *At1g04630-1* and *At1g04630-2* mutants were shorter and smaller than the wild type seedlings under normal growth conditions. During the whole vegetative growth stage, the *At1g04630-2* mutant plants were smaller than *At1g04630-1* (Fig 5D). Similar results were observed with regard to seed size. More specifically, the seeds of the two mutants were significantly smaller and poorly developed, especially for the mutant *At1g04630-2* (Fig 5D).

**Fig 6:**
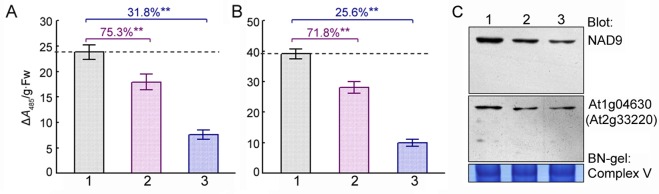
Reduced respiratory activity and assembly of mitochondrial complex I due to *At1g04630* mutant alleles. (A, B) The respiratory activities in the shoots (A) and roots (B). (C) Mitochondrial complex I assembly ability. 10-day-old seedlings were used. Lane 1, wild type (Col); lane 2, *At1g04630-1*; lane 3, *At1g04630-2*. Error bars indicate the sd (n≥15) from three independent experiments. *, *P*<0.05 and**, *P*<0.01; both compared with wild type plants.

The *At1g04630*-knockdown mutants possessed true leaves of different sizes and shapes compared to the wild type seedlings (S5 Fig). The leaves of the mutants were smaller (Fig 5F) and more rotund (smaller length:width ratio, Fig 5G). The petiole lengths of the mutants were also significantly shorter (Fig 5H). Considering the fifth leaf as an example, its lengths in *At1g04630-1* and *At1g04630-2* were only 69.7% and 43.9%, respectively, of that of the wild type (Fig 5H). Interestingly, a close-up view revealed that the leaf trichomes of both mutants were less numerous and were produced later compared with those of the wild type (S5 Fig and Fig 5E). Furthermore, significant declines in respiratory activities were detected in the shoots and roots of both mutant plants (Fig 6A and 6B). Western blot analysis also indicated that the amount of complex I was drastically decreased in both mutants, especially in the mutant *At1g04630-2* (Fig 6C), which showed decreases in both respiratory activity and complex I abundance of over 50%. Our data indicate that knockdown of *At1g04630*, which is a homologue of *GhMCS1* in Arabidopsis, leads not only to serious decreases in mitochondrial respiratory activity and complex I assembly but also to significant changes in mutant phenotypes, including fewer trichomes, smaller whole-plant architecture, and poorly developed seeds.

### Correlation between GhMCS1 level and respiratory activity during cotton fibre growth

**Fig 7:**
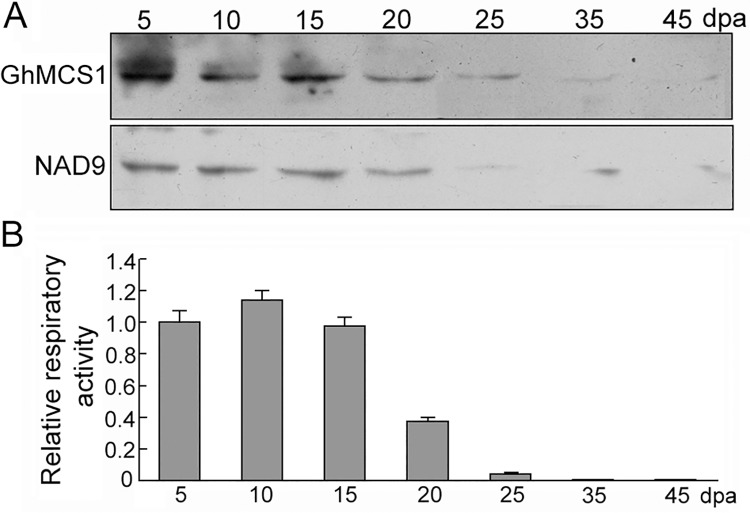
**Associations among GhMCS1 expression level, complex I intensity (A) and respiratory activity (B) during cotton fibre elongation and maturation.** 50 mg of total proteins from the fibre cells were separated by SDS-PAGE and subjected to Western blot analysis. Respiratory activities were assayed as described in “Experimental procedures”.

To reveal the correlations among GhMCS1 protein level, mitochondrial activity and fibre development, biochemical and cellular assays were performed to evaluate the developing cotton fibres. As shown in Fig 7A, the GhMCS1 protein accumulated at a relatively high level in the elongating fibres (5–15 dpa, elongation stage), which was in good agreement with the northern blot results (Fig 2B). Thereafter, GhMCS1 decreased to a trace level (25 dpa, early secondary cell wall synthesis stage), while no bands could be detected in the mature dehydrated fibres (45 dpa, dehydration stage). Very similar results were observed for the mitochondrial marker protein NAD9, which is a symbolic component of mitochondrial complex I (Fig 7A), suggesting that GhMCS1 expression is tightly controlled at the protein level during the process of cotton fibre development and growth. TTC assays also showed a drastic decline in respiratory activity from 10 dpa to 25 dpa along with a decrease in the GhMCS1 protein level (Fig 7B). Not surprisingly, no activity could be detected in the dehydrated fibres (45 dpa), indicating an important correlation of respiratory activity with cotton fibre growth. Taken together, these data indicate that the GhMCS1 protein level is closely correlated with the respiratory activity of cotton fibres and also with fibre growth.

## Discussions

In this study, a cotton *GhMCS1* gene encoding a subunit of mitochondrial complex I was characterised. GhMCS1 shares high sequence similarities with GRIM-19 proteins, which have been extensively studied in animals (Fig 1). To determine whether GhMCS1 functions as a bona fide subunit of complex I in cotton, we performed a series of biochemical and molecular assays. Using BN-PAGE and Western blot assays, GhMCS1 was found to be localised to the mitochondria and to colocalise with OXPHOS complex I (Fig 3). Inducible overexpression of *GhMCS1* in Arabidopsis resulted in enhancements in respiratory activity and complex I assembly (Fig 4). Although loss-of-function analysis of *GhMCS1* was not performed due to difficulties involved in the production of transgenic cotton, Arabidopsis mutants with knockdown of *At1g04630* (the closest orthologue of *GhMCS1*) were identified. The *At1g04630* mutation resulted in a significant reduction in both respiratory activity and complex I assembly (Fig 6). Obviously, GhMCS1 functions as a critical subunit involved in mitochondrial complex I assembly and in cotton fibre growth.

As mentioned above, GRIM-19 proteins have been detected in animal mitochondrial complex I and are essential for complex I assembly and electron transfer activity [8, 50]. More importantly, the functional domains in GRIM-19 proteins have also been defined in mammalian cells. As previously reported, the N-terminal domain (1–60 aa) is required for mitochondrial localisation and complex I incorporation, the middle region (70–100 aa) is essential for electron transfer activity of complex I (Δ*Ψ*m), and the 10-aa C-terminal region may promote GRIM-19-mediated assembly of complex I [51]. Based on sequence alignment analysis, GhMCS1 as well as its Arabidopsis orthologue At1g04630 possess conserved middle regions (Fig 1), indicating their potential roles in maintaining Δ*Ψ*m. However, these plant orthologues of GRIM-19 lack C-terminal regions (Fig 1), suggesting a specific assembly mechanism of mitochondrial complex I mediated by GhMCS1 and its orthologues in plants. Further research is required to elucidate these mechanisms.

*GhMCS1* transcripts were detected to be enriched in developing fibres during the elongation stage (Fig 2B). Both Arabidopsis trichomes and cotton fibres shared the similar process of cell elongation. The coss-species gene over-expression and complementation experiments have identified the common transcriptional regulatory network between them [52]. Additionally, the xylems can also serve as the model for the late stages of cotton fiber development [53]. In Arabidopsis, the *GhMCS1* promoter may drive GUS reporter gene expression in the trichomes and vascular tissues of leaves (Fig 2C). Besides, the *At1g04630* mutant alleles produced less trichomes than the wild type plants (Fig 5). Furthermore, a close correlation was found among GhMCS1 protein level, respiratory activity and fibre developmental phase (Fig 7), indicating that GhMCS1 may participate in cotton fibre development by regulating mitochondrial activity. These results suggested that GhMCS1 is involved in cotton fibre growth and development. However, further evidence is necessary to elucidate the detailed underlying mechanisms.

Pang *et al*. have reported that an additional mitochondrial protein, the δ1 subunit of ATP synthase (GhATPδ1), is related to cotton fibre growth [54]. *GhATPδ1* expression is significantly up-regulated during fibre cell elongation, showing expression patterns similar to those of *GhMCS1*. During fibre cell wall formation and elongation, high energy is required. In fibre cells, *GhATPδ1* expression may result in an elevated ATP:ADP ratio, while exogenous application of inhibitors of ATP synthase in ovule culture media may lower the ATP:ADP ratio, resulting in shorter fibres. In mitochondria, concomitant proton translocation by the membrane arm of complex I is coupled to OXPHOS, generating ATP [55]. This is the major source of intracellular ATP, especially in non-photosynthetic tissues. Furthermore, the transport of H^+^ and the exchange of ATP/ADP across the inner membrane are driven by Δ*Ψ*m. As discussed previously, GhMCS1 may play an essential role in maintaining Δ*Ψ*m [51]. Therefore, GhMCS1 may function to limiting mitochondrial ATP generation. During cotton fibre growth, GhMCS1 expression may ensure for the precise assembly of mitochondrial complex I and maintenance of Δ*Ψ*m across the inner membrane, leading to coupled ATP synthesis, although further evidence is necessary to verify this hypothesis. The Δ*Ψ*m value maintained by GhMCS1 and the resulting ΔpH can also drive the export of organic acids from the mitochondrial matrix to the cytosol. Organic acids, including citrate, succinate, malate, and oxaloacetate, are metabolites of the TCA cycle. They can be translocated across the inner mitochondrial membrane by a series of specific transporters. During cotton fibre elongation, these organic acids can function as osmotic regulators to maintain cellular turgor pressure, leading to unidirectional outgrowth from the epidermis of the developing ovule [56, 57]. To further reveal the relationship of mitochondrial complex I and respiratory activity in cotton fibre growth, the effects of respiratory inhibitors, such as rotenone and piericidin A, would be detected in cotton ovules culture.

In addition to its potential roles in cotton fibre growth, other functions of GhMCS1 and its orthologs have not been excluded. As described in this study, the Arabidopsis mutants with knockdown of *At1g04630*, the *GhMCS1* ortholog, showed smaller seedling phenotypes and seed sizes. Additionally, smaller and rounder leaves and shorter petioles were found in these mutants (Fig 5, S4 Fig). In another study involving a large-scale mutant screen of Ds transposon insertion lines, *At1g04630* was identified as *AtMEE4* (*m**aternal* *e**ffect* *e**mbryo arrest 4*), and its disruption resulted in the arrest of endosperm development at the one-cell zygotic stage [58]. Similarly, in mice, GRIM-19 knockout has been shown to cause early embryonic lethality [8], and its knockdown in *Xenopus* has been demonstrated to lead to failure of early heart development [59]. These results indicate that GhMCS1 and its orthologs might also be critical for both seedling morphogenesis and embryonic development in plant. However, whether these functions are associated with their roles in mitochondrial complex I assembly are largely unknown.

## Conclusions

In conclusion, the current work provides a comprehensive description of the *GhMCS1* gene, including its expression pattern, subcellular localisation and potential roles in cotton fibre growth. These results collectively suggest that GhMCS1 is a conserved subunit of mitochondrial complex I in cotton. Both gain-of-function and loss-of-function transgenic assays suggest that GhMCS1 can facilitate the assembly of complex I and efficiently maintain the integrity and activity of mitochondria. We also have shown that GhMCS1 is associated with cotton fibre growth. Further investigations are expected to reveal the detailed mechanisms of GhMCS1 in complex I assembly and cotton fibre growth.

## Supporting Information

S1 Fig The nucleic acid sequence of the *GhMCS1* gene and the deduced amino acid sequence. The nucleotide “A” in the translation initiation codon (ATG) is designated as +1. The exon sequences are bolded.
(TIF)

S2 Fig **Multiple copies of *GhMCS1* in tetraploid cotton subgenome (AADD) (A) and their sequence alignment with GhMCS1 (B).** The theoretical molecular weight (MW) and isoelectric point (p*I*) were calculated by ExPASy (http://cn.expasy.org/tools). The sequences were aligned with ClustalX using the default parameters, and the box shading was created by BOXSHADE 3.21.
(TIF)

S3 Fig DEX-inducible expression of *GhMCS1* in transgenic Arabidopsis. (A) Schematic diagrams of the DEX-inducible vector constructs. LB, left T-DNA border; 6×UAS, glucocorticoid-regulated transcription factor (GVG)-regulated promoter; 3A-ter, pea rbcS-3A terminator; NOS-ter, nopaline synthetase terminator; HPT II (Hyg^r^), hygromycin phosphotransferase II coding sequence; E9-ter, pea rbcS-E9 terminator; GR-HBD-VP16GAL, the transcription activation domain of VP16 and the regulatory region of the human glucocorticoid receptor; CaMV 35S-pro, cauliflower mosaic virus (CaMV) 35S promoter; RB, right T-DNA border.(B, C) Detection of *GhMCS1* transcripts and proteins in different transgenic lines by RT-PCR (B) and Western blot (C) analysis, respectively. 10-d-old seedlings germinated and grown in the absence of DEX were transferred onto the inductive medium containing 10 μM DEX and cultured at 23°C for 3 days. The seedlings without DEX-induction were used as control. Vector, Arabidopsis seedlings carrying the empty pTA7002 as the negative control. (D) Dose dependency of DEX-induced GhMCS1 protein level in transgenic lines. 10-d-old seedlings of different lines grown on non-inductive medium were transferred onto medium containing each concentration of DEX and cultured at 23°C for 3 days. (E) Mitochondrial localization of accumulated GhMCS1 protein under various concentrations of DEX-induction.
(TIF)

S4 Fig RT-PCR analysis of transcripts of *GhMCS1* homologues (*At1g04630* and *At2g33220*) in Arabidopsis plants. Si, siliques; Sd, seeds; Fl, flowers; Rt, roots; Lf, leaves; St, stems. The PCR was performed with primers aligned to both *At1g04630* and *At2g33220*. Arabidopsis *Actin2* was amplified as a control.
(TIF)

S5 Fig Representative images of leaves in the wild types (Col) and *At1g04630* mutants (*At1g04630-1; At1g04630-2*), with the close-up views (leaf No. 7) shown at right. (TIF)
