# Supplementary figures and images for: GhMCS1, the Cotton Orthologue of Human GRIM-19, Is a Subunit of Mitochondrial Complex I and Associated with Cotton Fibre Growth
Source: PLoS One. 2016 Sep 15;11(9):e0162928. doi: 10.1371/journal.pone.0162928 (PMC5025012; doi:10.1371/journal.pone.0162928)

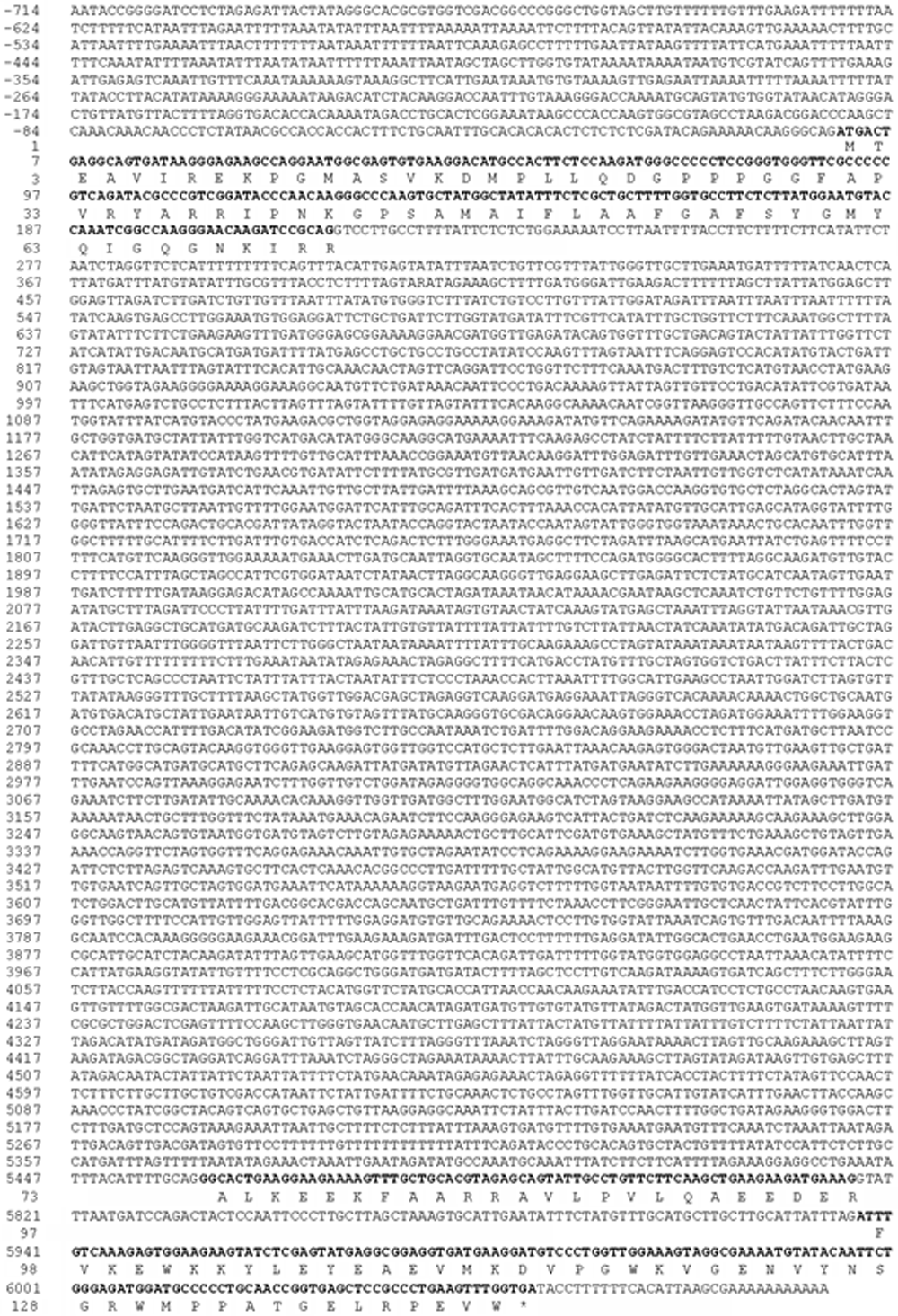

Supplement: S1 Fig — The nucleotide “A” in the translation initiation codon (ATG) is designated as +1. The exon sequences are bolded. (TIF) [file pone.0162928.s001.tif]

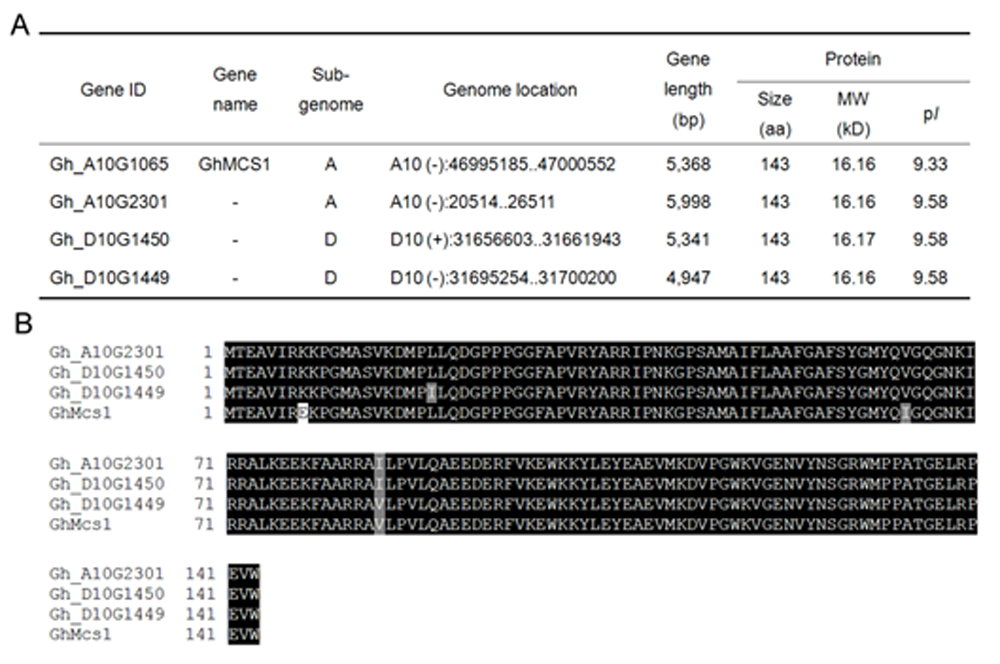

Supplement: S2 Fig — Multiple copies of GhMCS1 in tetraploid cotton subgenome (AADD) (A) and their sequence alignment with GhMCS1 (B). The theoretical molecular weight (MW) and isoelectric point (pI) were calculated by ExPASy (http://cn.expasy.org/tools). The sequences were aligned with ClustalX using the default parameters, and the box shading was created by BOXSHADE 3.21. (TIF) [file pone.0162928.s002.tif]

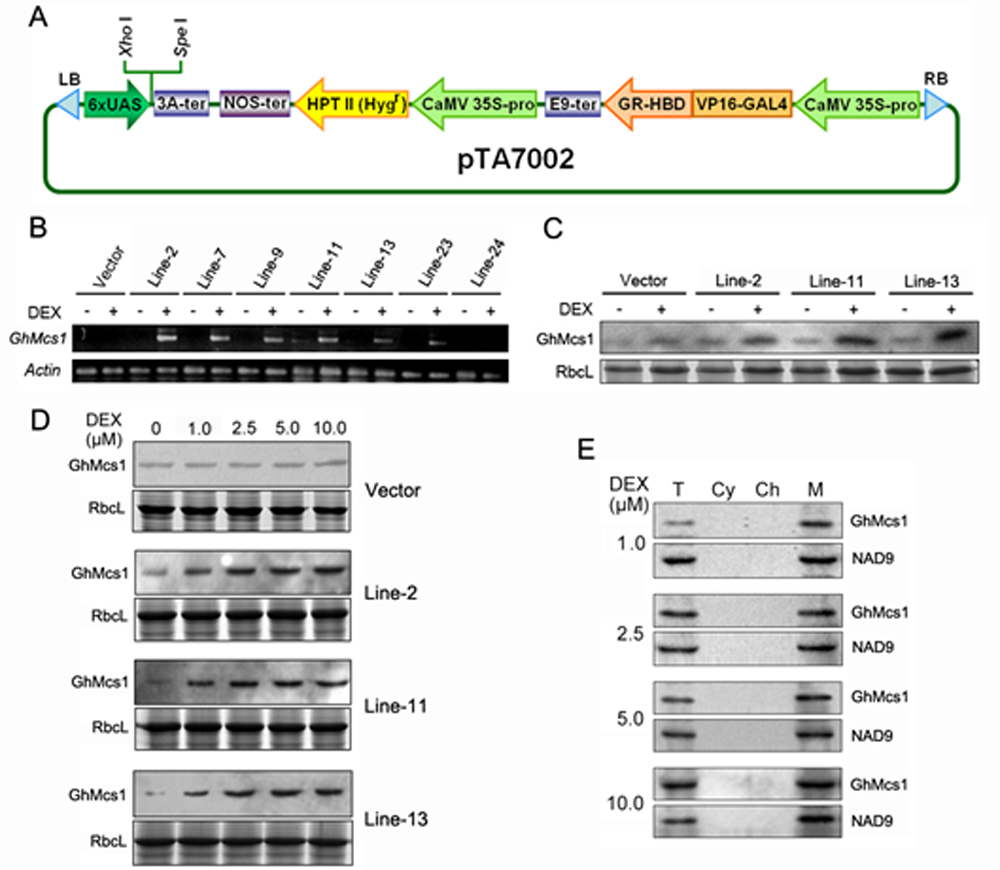

Supplement: S3 Fig — (A) Schematic diagrams of the DEX-inducible vector constructs. LB, left T-DNA border; 6×UAS, glucocorticoid-regulated transcription factor (GVG)-regulated promoter; 3A-ter, pea rbcS-3A terminator; NOS-ter, nopaline synthetase terminator; HPT II (Hygr), hygromycin phosphotransferase II coding sequence; E9-ter, pea rbcS-E9 terminator; GR-HBD-VP16GAL, the transcription activation domain of VP16 and the regulatory region of the human glucocorticoid receptor; CaMV 35S-pro, cauliflower mosaic virus (CaMV) 35S promoter; RB, right T-DNA border.(B, C) Detection of GhMCS1 transcripts and proteins in different transgenic lines by RT-PCR (B) and Western blot (C) analysis, respectively. 10-d-old seedlings germinated and grown in the absence of DEX were transferred onto the inductive medium containing 10 μM DEX and cultured at 23°C for 3 days. The seedlings without DEX-induction were used as control. Vector, Arabidopsis seedlings carrying the empty pTA7002 as the negative control. (D) Dose dependency of DEX-induced GhMCS1 protein level in transgenic lines. 10-d-old seedlings of different lines grown on non-inductive medium were transferred onto medium containing each concentration of DEX and cultured at 23°C for 3 days. (E) Mitochondrial localization of accumulated GhMCS1 protein under various concentrations of DEX-induction. (TIF) [file pone.0162928.s003.tif]

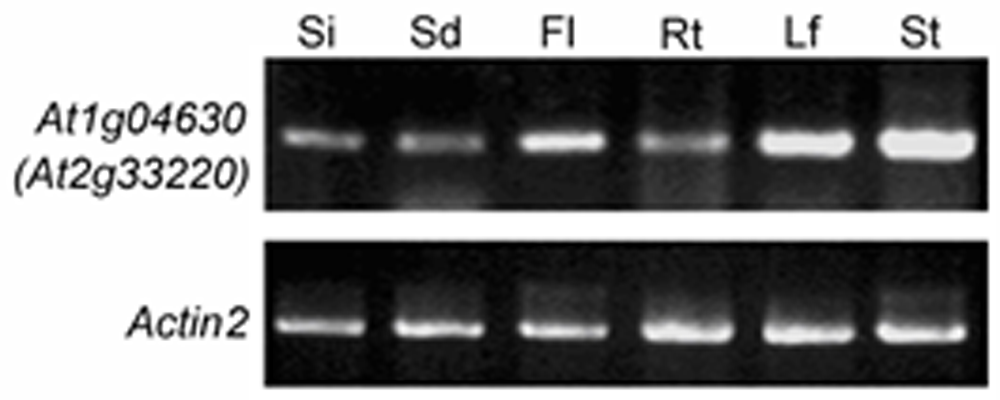

Supplement: S4 Fig — Si, siliques; Sd, seeds; Fl, flowers; Rt, roots; Lf, leaves; St, stems. The PCR was performed with primers aligned to both At1g04630 and At2g33220. Arabidopsis Actin2 was amplified as a control. (TIF) [file pone.0162928.s004.tif]

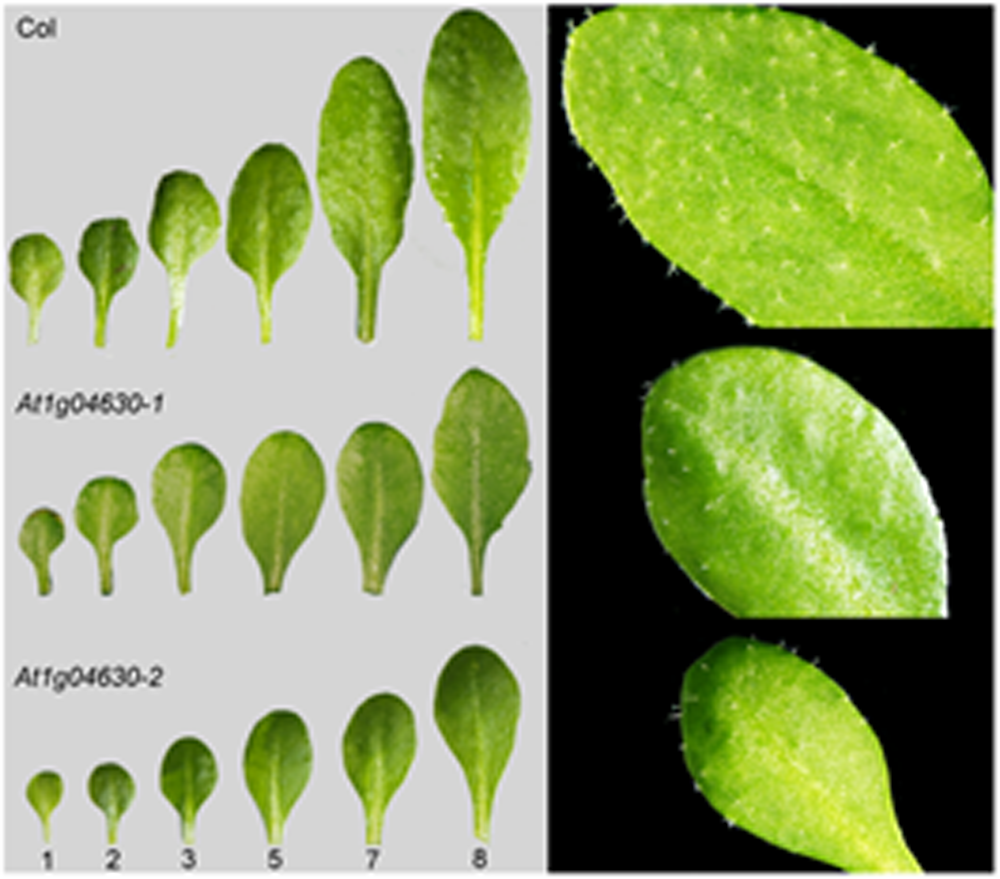

Supplement: S5 Fig — (TIF) [file pone.0162928.s005.tif]
